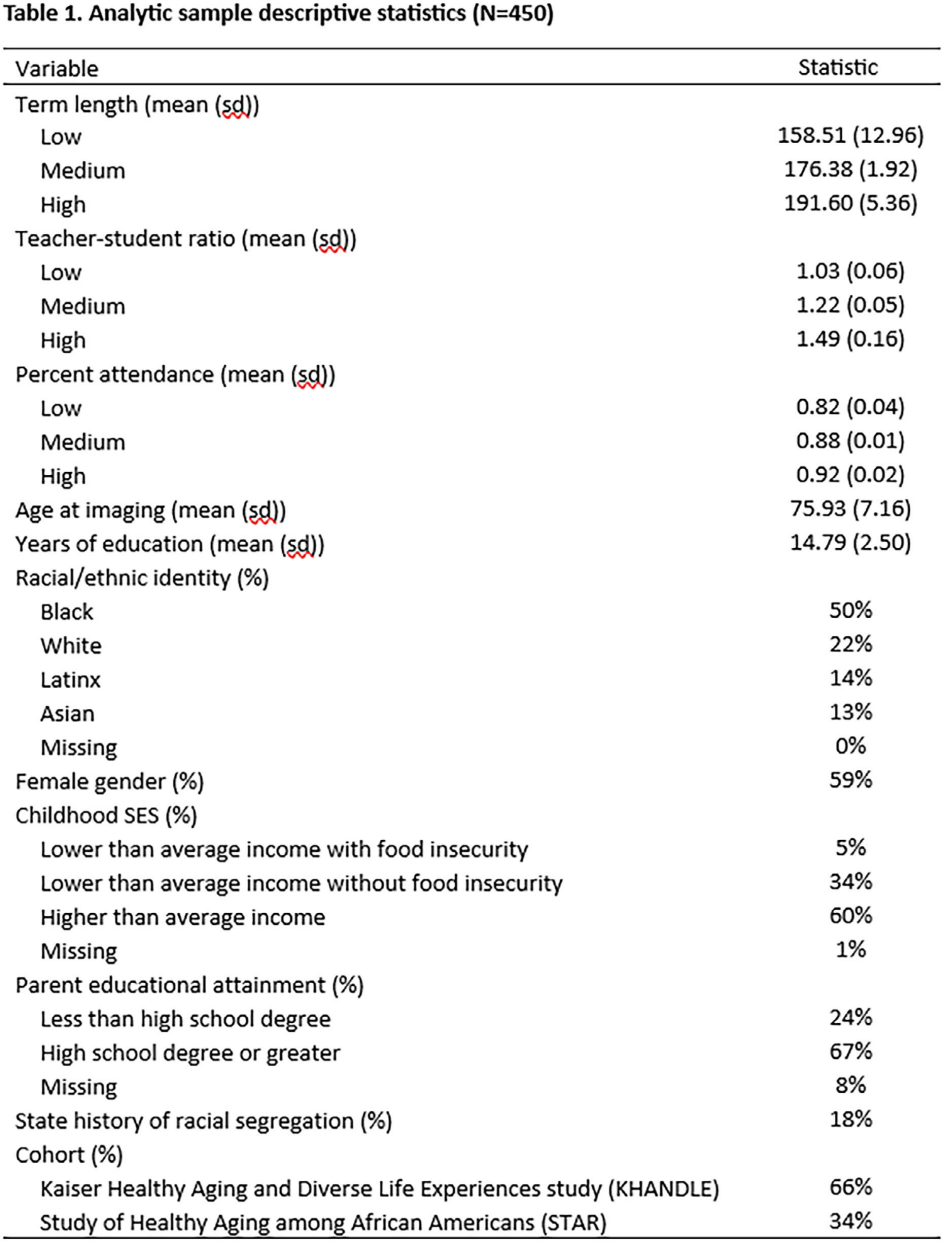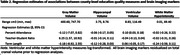# County‐level indicators of education quality and brain imaging markers of neurodegeneration and vascular injury among racially and ethnically diverse middle‐aged and older adults

**DOI:** 10.1002/alz.093321

**Published:** 2025-01-09

**Authors:** Joseph Roscoe, Rachel A. Whitmer, Sirena Gutierrez, Chloe W. Eng, M. Maria Glymour, Charles Decarli, Lisa L. Barnes, Brandon E Gavett, Paola Gilsanz

**Affiliations:** ^1^ Kaiser Permanente Northern California Division of Research, Oakland, CA USA; ^2^ University of California, Davis, Davis, CA USA; ^3^ University of California San Francisco, San Francisco, CA USA; ^4^ Stanford University, Stanford, CA USA; ^5^ Boston University School of Public Health, Boston, MA USA; ^6^ Rush Alzheimer’s Disease Center, Chicago, IL USA; ^7^ University of California Davis, Sacramento, CA USA

## Abstract

**Background:**

Early‐life education quality has been associated with dementia risk and late‐life cognitive functioning. However, the association between education quality and neuroimaging outcomes remains unclear.

**Methods:**

These analyses utilized data from 450 participants in two harmonized cohorts of racially and ethnically diverse adults aged 50 years and older (KHANDLE and STAR) who completed brain Magnetic Resonance Imaging and whose self‐reported school location at 9^th^ grade could be linked to historical educational quality data from the National Center for Education Statistics. Three county‐level indicators of 9^th^ grade education quality were categorized into tertiles based on the pooled dataset and treated as ordinal variables with the lowest tertile serving as the reference: percent attendance, teacher‐student ratio (number of teachers per 30 students), and term length. Measures of gray matter volume, hippocampal volume, white matter hyperintensity volume (log‐transformed), and ventricular volume (log‐transformed) were residualized on total cranial volume. Linear regression models with robust standard errors estimated associations between education quality measures and each imaging marker, adjusting for age at imaging, demographics, childhood socioeconomic status, study cohort, and history of racial segregation in state of 9^th^ grade education. Sensitivity analyses examined each education quality measures separately, adjusting for covariates.

**Results:**

Higher teacher‐student ratio was associated with greater gray matter volume (β = 6.25, 95% CI: 1.57, 10.92) but was not associated hippocampal volume (β = ‐0.01, 95% CI: ‐0.14, 0.11), ventricular volume (β = ‐0.04, 95% CI: ‐0.13, 0.04), or white matter hyperintensity (β = ‐0.02, 95% CI: ‐0.31, 0.26) (Table 2). Higher percent attendance was associated with greater ventricular volume (β = 0.10, 95% CI: 0.03, 0.18) but was not associated with gray matter volume (β = ‐3.12, 95% CI: ‐7.07, ‐0.82), hippocampal volume (β = ‐0.06, 95% CI: (‐0.17, 0.05), or white matter hyperintensity (β = ‐0.02, 95% CI: ‐0.26, 0.22). Term length was not associated with any marker of neurodegeneration or vascular brain injury. Results were similar when examining each education quality measure separately.

**Conclusions:**

County‐level teacher‐student ratio and percent attendance, markers of education quality, had qualitatively different associations with imaging markers of neurodegeneration. Additional studies in larger samples are needed to confirm and better understand underlying reasons for these potential differences.